# SOX4 interacts with plakoglobin in a Wnt3a-dependent manner in prostate cancer cells

**DOI:** 10.1186/1471-2121-12-50

**Published:** 2011-11-19

**Authors:** Yu-Heng Lai, Jessica Cheng, Dongmei Cheng, Mattie E Feasel, Kyle D Beste, Junmin Peng, Asma Nusrat, Carlos S Moreno

**Affiliations:** 1 Program in Genetics & Molecular Biology, Emory University, Atlanta, GA 30322, USA; 2 Department of Pathology & Laboratory Medicine, Emory University School of Medicine, Atlanta, GA 30322, USA; 3 Department of Human Genetics, Emory Proteomics Service Center, Emory University, Atlanta, GA 30322, USA; 4 Winship Cancer Institute, Emory University School of Medicine, Atlanta, GA 30322, USA; 5 Department of Structural Biology, St. Jude Proteomics Facility, St. Jude Children's Research Hospital, Memphis, TN 38105, USA; 6 Department of Developmental Neurobiology, St. Jude Proteomics Facility, St. Jude Children's Research Hospital, Memphis, TN 38105, USA

## Abstract

**Background:**

SOX4 is a developmental transcription factor that is required for differentiation and proliferation in multiple tissues. SOX4 is overexpressed in many human malignancies, but the precise role of SOX4 in cancer progression is still not well understood. Thus, the identification of additional SOX4 binding partners is essential for elucidating the mechanism of SOX4-mediated effects in cancer progression.

**Results:**

Here, we have adapted a one-step affinity purification method that enables rapid purification of SOX4 complexes via intracellular biotinylation of the amino-terminus of SOX4 to perform large-scale proteomics analysis. We have discovered that junction plakoglobin (JUP) interacts with SOX4 in both the cytosol and the nucleus and the interaction between SOX4 and plakoglobin is significantly increased when prostate and breast cancer cells are stimulated with WNT3A. Interactions between SOX4 and plakoglobin were further enhanced by the nuclear export inhibitor leptomycin B (LMB), suggesting that plakoglobin promotes nuclear export of SOX4. The SOX4-plakoglobin complex affected the expression of Wnt pathway target genes and SOX4 downstream targets, such as *AXIN2*, *DICER1*, and *DHX9*. In addition, SOX4 DNA binding activity to the promoters of *DICER1*, *AXIN2*, *DHX9 *and *SOX4 *itself was reduced by conditions that promote SOX4-plakoglobin complex formation. Conditions that enhanced SOX4-plakoglobin interactions resulted in reduced transcriptional activity of β-catenin luciferase reporters.

**Conclusions:**

These data suggest that this newly identified interaction between SOX4 and plakoglobin is inhibitory and provides new insights into the role of SOX4 in key pathways in cell proliferation, development, and cancer progression.

## Background

The sex-determining region Y (SRY) box, or SOX, family consists of 20 highly conserved transcription factors in humans that play important roles in development [1]. SOX4 is a 47-kDa protein that is encoded by a single exon gene and contains a conserved signature sequence in the high-mobility group (HMG) DNA-binding domain (DBD) related to the TCF/LEF family of transcription factors. The key effector of the canonical Wnt pathway, β-catenin, forms complexes with TCF/LEF HMG box factors and stimulates the transcription of Wnt downstream targets. Many studies have shown that β-catenin is regulated at several levels in cancer progression, but less is known about the regulation of the TCF/LEF transcription factor family. Structurally related to TCF/LEFs, several members of the SOX family, SOX17, SOX7, SOX9, and SOX4 have been implicated in regulating β-catenin activity [2-4]. Gain- and loss-of-function analyses have demonstrated that SOX17 and SOX7 proteins antagonize the Wnt pathway by competing with TCFs for β-catenin binding while SOX4 may function to stabilize β-catenin protein to help activate expression of target genes and promote cellular proliferation [5]. Although these findings have implicated how SOX proteins regulate the transcriptional output of Wnt pathway, the precise role of SOX4 in the Wnt pathway remains unclear.

In humans, tight regulation of the levels of transcriptional factors is crucial to maintain tissue homoeostasis and many have been found to be oncogenic when their expression is deregulated or when their activity is functionally altered [6]. For example, SOX4 is overexpressed in several cancers, such as bladder cancer, in which it is 5-fold upregulated compared with normal tissues [7]. SOX4 is also upregulated at the mRNA and protein level in prostate cancer and this upregulation is correlated with higher Gleason score or tumor grade [8]. In addition, SOX4 is overexpressed in leukemias, melanomas, glioblastomas, medulloblastomas [9], and lung cancer [10]. SOX4 is also overexpressed in endometrial cancer through methylation-mediated silencing of miR-129-2 [11]. Consistent with the concept that SOX4 is an oncogene, three independent studies searching for oncogenes have found SOX4 to be one of the most common retroviral integration sites, resulting in increased mRNA [12-14].

Junction plakoglobin (JUP), also known as γ-catenin, is a major component of the submembrane of adherens junctions and desmosomes in mammalian cells [15]. It is closely related to the Drosophila segment polarity gene armadillo, which has a role in the transduction of transmembrane signals that regulate cell fate [16,17]. Plakoglobin shares more than 76% homology with β-catenin, contains a central armadillo repeat domain flanked by the carboxyl and amino terminal domains, and functions in cell-cell junctions, along with β-catenin and α-catenin when coupled with cadherins [18]. While it is known that β-catenin is essential in the Wnt signaling cascade, plakoglobin also binds to TCF/LEF factors, and has lower TCF/LEF-dependent transcriptional activity compared to β-catenin when endogenous β-catenin is depleted [19,20].

Mass spectrometry is a highly sensitive technique that enables the rapid identification of proteins and also protein-protein interactions from a variety of biological samples. When combined with affinity purification, whole or targeted protein interaction networks can be elucidated [21]. To investigate the cellular functions of SOX4, we have developed a one-step affinity purification method that enables rapid purification of SOX4 complexes in LNCaP cells, a prostate cancer cell line. Here, in this study, we identified proteins that interact with SOX4 in LNCaP cells by liquid chromatography-tandem mass spectrometry (LC-MS/MS), including plakoglobin. This novel interaction between SOX4 and plakoglobin may provide insights into the role of SOX4 in key pathways in cell proliferation, development, and cancer progression.

## Methods

### Reagents and cell culture

LNCaP, PC3M, and MDA-MB-231 cells were cultured as described [22] by American Type Culture Collection except using T-medium (Invitrogen) for LNCaP cells. HA-tagged SOX4 was cloned into pHR-UBQ-IRES-eYFP-ΔU3 lentiviral vector (gift from Dr. Hihn Ly, Emory University), and stable cells were isolated, as previously described [23]. Recombinant WNT3A was purchased from R & D Systems (5036-WNP) and reconstituted in 0.1% BSA/PBS prior to use. Nuclear export inhinbitor leptomycin b (LMB) was purchased from Sigma (L2913). Cells were treated for 24 hrs with 100 ng/ml WNT3A, 20 μM LMB, or both unless otherwise noted.

### Biotinlyated HA-tagged SOX4 expression construct

The pREP4-BLRPwt-IRES-BirA-XL9 plasmid was a gift from Dr. Jeremy Boss (Emory University). The pcDNA3.1-HisA-HASOX4 was constructed as described [8]. To generate pREP4-BLRPwt-HASOX4-IRES-BirA-XL9, HA-tagged SOX4 from pcDNA3.1-HisA-HASOX4 was excised at KpnI and XbaI sites and overhangs were filled in with Klenow fragment. Filled-in HA-SOX4 was then blunt ligated into the filled-in NotI site of pREP4-BLRPwt-IRES-BirA-XL9.

### Purification of biotinlyated HA-tagged SOX4

The pREP4-BLRPwt-HASOX4-IRES-BirA-XL9 and control empty vector were transfected into two 90% confluent 100 mm dishes of LNCaP cells respectively. Forty-eight hours post-transfection, the plates were then placed on ice and the cells were washed twice with ice-cold PBS, and lysed in 1 ml/plate with ice-cold IP lysis buffer (0.137 M NaCl, 0.02 M Tris pH8.0, 10% glycerol, 1% NP-40) supplemented with protease inhibitors, and harvested by scraping. Biotinlyated HA-tagged SOX4 complexes were purified by incubating with 50 ul slurry of Dynabeads^® ^M280 Streptavidin (Invitrogen) at 4°C for 2 hrs. The beads ware washed 3 times with IP lysis buffer and eluted by boiling in Laemmli sample buffer.

### Sample preparation for mass spectrometry

Five percent of the proteins resulting from the purification were subject to 4-15% gradient SDS-PAGE and silver staining to analyze sample purity. The rest of the purified proteins were subjected to 4-15% gradient SDS-PAGE and concentrated on a very short distance (~2 mm long), and the protein in those bands were excised for in-gel digestion and Liquid chromatography coupled with tandem mass spectrometry based on an optimized protocol [21].

### Co-immunoprecipitation and Western blot

Cells were washed twice with ice-cold PBS, lysed in 1 ml/plate with ice-cold IP lysis buffer supplemented with protease inhibitors, and harvested by scraping. The whole cell lysates were pre-cleared and then incubated with 25 ul slurry of Dynabeads^® ^M280 Streptavidin or protein G (Invitrogen) at 4°C for 2 hrs. The beads were washed 3 times with IP lysis buffer and eluted by boiling in Laemmli sample buffer before running SDS-PAGE. Western blots were performed as previously described [23]. Antibodies to plakoglobin (13-8500, Invitrogen) and hemagglutinin (HA12CA5) were used in immunoprecipitations (IPs). Antibodies to plakoglobin (610253, BD Biosciences), SOX4 (LS-B3520, LifeSpan Biosciences), β-actin (3700S, Cell Signaling), and HA 16B12 (AFC-101P-1000, Covance Research Products) were used in Western blot.

### Confocal microscopy

Sub-confluent LNCaP HASOX4 stable cells were grown on glass cover slips, and serum starved for 24 hrs with 0.5% FBS (fetal bovine serum) T-Medium before treating with 100 ng/ml WNT3A, 20 μM LMB, or both for another 24 hrs. Cells were washed three times with Hank's Balanced Salt Solution (HBSS^+^) and fixed with 3.7% paraformaldehyde for 20 min at room temperature. Cells were permeablized with 100% ethanol for 20 min at -20°C and blocked in 3% BSA in HBSS^+ ^for 1hr. anti-HA 16B12 (1:500 dilution) and plakoglobin antibodies (1:50 dilution) (sc-H80, Santa Cruz Biotechnology) were used as primary antibodies and were diluted in blocking buffer and incubated for 1 hr. Then cells were washed in HBSS^+ ^and incubated in fluorescence-labeled secondary antibodies for 1hr at room temperature. Cells were washed in HBSS^+ ^and then stained with Topro (T3605, Invitrogen) for 5 min at room temperature. Labeled cells were then washed in HBSS^+ ^and mounted in p-phenylene diamine antifade agent. Confocal fluorescence images were captured using a laser-scanning microscope.

### Cell fractionation into nuclear and cytosolic lysates

One 100-mm dish of LNCaP HASOX4 stable cells was grown to 80-90% confluency and serum starved for 24 hrs with 0.5% FBS T-Medium before treating with 100 ng/ml WNT3A, 20 μM LMB, or both for another 24 hrs. Crude subcellular fractionation was performed as previously described using digitonin, NP40, and RIPA lysis methods [24].

### Quantitative real-time PCR

Ninety-percent confluent cells were harvested using the RNeasy kit (Qiagen), and reverse transcription was performed using iScript cDNA Synthesis Kit (Bio-Rad Laboratories). Quantitative real-time PCR (qPCR) was performed using iQ SYBR Green Supermix (Bio-Rad Laboratories) on a Bio-Rad iCycler using 18s or β-actin as a control, and data were analyzed using the δCt method [25].

### Chromatin immunoprecipitation (ChIP) assay

One 100-mm dish of LNCaP HASOX4 stable cells was grown to 80-90% confluency. Cells were fixed with 1% formaldehyde, then lysed, and sonicated as described [26]. Sonicated chromatin was precleared and then immunoprecipitated with 4 μg of anti-HA 12CA5 ascites or mouse IgG overnight and immunoprecipitated by Dynabeads^® ^Protein G (10004D, Invitrogen) for 2 hrs at 4°C. Beads were washed and eluted as described [26]. ChIP DNA was purified and then subjected to PCR amplification.

### Luciferase reporter assay

Cells grown in twelve-well tissue culture plates were transfected with 0.5 μg of either TOP-flash or FOP-flash with 0.04 μg of TK-Renilla control vector (Promega). At 6 hrs post-transfection, cells were placed in 0.5% FBS medium for recovery and serum starvation. At 24 hrs post-transfection, cells were treated with WNT3A and LMB, or both. Reporter gene activity was measured in a TD-20/20 luminometer (Turner Design) with the DLR Luciferase Assay System (Promega) after 48 hrs and was normalized for transfection efficiency by measuring Renilla luciferase activity.

### siRNA transfection

The siRNA sequence for plakoglobin AGTCGGCCATTGTGCATCT was targeted at the 5' end of the gene lacking of homology with other catenin members [27] (Dharmacon RNA Technologies). LNCaP HASOX4 cells were transfected using Lipofectamine 2000 (Invitrogen) with plakoglobin or scramble siRNAs at final concentration of 200 nM. At 6 hrs post-transfection, cells were placed in 0.5% FBS medium for recovery and serum starvation. At 24 hrs post-transfection, cells were treated with WNT3A, LMB, or both for Western blot analysis or chromatin immunoprecipitation assay.

## Results

### Identification of proteins that interact with SOX4

**Figure 1:**
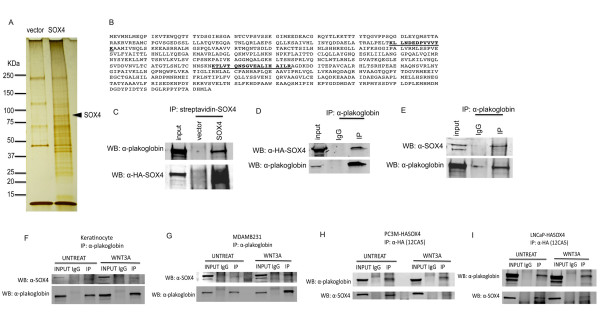
**Plakoglobin binds to SOX4 in LNCaP cells**. *A*. Whole cell lysates prepared from LNCaP cells transfected with pREP4-BLRPwt-HASOX4-IRES-BirA-XL9 or vector only were purified using an streptavidin-magnetic beads, and affinity-purified Vector- and SOX4-complexes were visualized by silver staining. SOX4 protein is indicated by an arrow. The molecular weight markers are as shown on the left. *B*. Amino acid sequence of plakoglobin with trypsinized peptides from LC-MS/MS analysis indicated in bold and underlined. *C*, pREP4-BLRPwt-HASOX4-IRES-BirA-XL9 and vector control transfected LNCaP cells. SOX4-associated plakoglobin were analyzed by Western blot with antibodies indicated. Equivalent amounts of the Vector-purified fractions were used to confirm specificity. 5% of transfected LNCaP whole cell lysate was used as Input. *D*, endogenous plakoglobin IPs were analyzed for transfected SOX4 by Western blot with anti-HA 16B12 mAb. *E*, endogenous plakoglobin IPs were analyzed for endogenous SOX4 in untransfected LNCaP cells. F, G, endogenous plakoglobin IPs were analyzed for endogenous SOX4 in primary human keratinocytes and breast cancer cell line, MDA-MB-231, respectively. H, I, HASOX4 IPs were analyzed for endogenous plakoglobin in stably-expressed SOX4 PC3M and LNCaP cell lines. F-I, whole cell lysates were harvested and treated with DNase I for 1 hr at room temperature prior to immunoprecipitation.

To identify proteins that stably interact with SOX4, we developed a one-step affinity purification method that allows for rapid purification of SOX4 complexes. The pREP4-BLRPwt-IRES-BirA-XL9 plasmid contains the *birA *gene of *E. coli *that encodes a biotin holoenzyme synthetase [28]. We cloned the human SOX4 gene into this vector to generate an amino-terminal fusion to a BirA recognition sequence to produce transiently expressed, intracellularly biotinylated SOX4 protein in LNCaP prostate cancer cells, and purified SOX4 complexes with streptavidin-linked magnetic beads to perform large scale proteomics analysis. Protein quantity and purity were checked by SDS-PAGE analysis and silver staining prior to mass spectrometry analysis (Figure 1A).

### Plakoglobin interacts with SOX4

LC-MS/MS analysis identified junction plakoglobin (JUP) as a SOX4 binding protein via two trypsinized fragments that perfectly matched to human plakoglobin sequences (Figure 1B). To confirm this interaction, we first repeated the transient transfection of the pREP4-BLRPwt-IRES-BirA-XL9-HASOX4 or vector control plasmid into LNCaP cells, and performed streptavidin-magnetic bead based purification followed by anti-plakoglobin (JUP) immunoblotting. As expected, the result of the immunoprecipitation (IP) and western blot validated the interaction between SOX4 and plakoglobin (Figure 1C). Furthermore, we performed a reverse-IP immunoprecipitating endogenous plakoglobin and probing for transfected HA-SOX4 (Figure 1D) and endogenous SOX4 in untransfected LNCaP cells (Figure 1E). To determine that the interactions were not cell-line specific or DNA-dependent, we repeated the immunoprecipitations in primary human keratinocytes, MDA-MB-231 breast cancer cells, and PC3M prostate cancer cells (Figure 1F-I) following DNase I digestion of whole cell lysates. Taken together, these data demonstrate that SOX4 directly interacts with plakoglobin and that plakoglobin is a novel SOX4 binding partner.

### Interaction between SOX4 and plakoglobin in the nucleus responds to Wnt signaling

**Figure 2:**
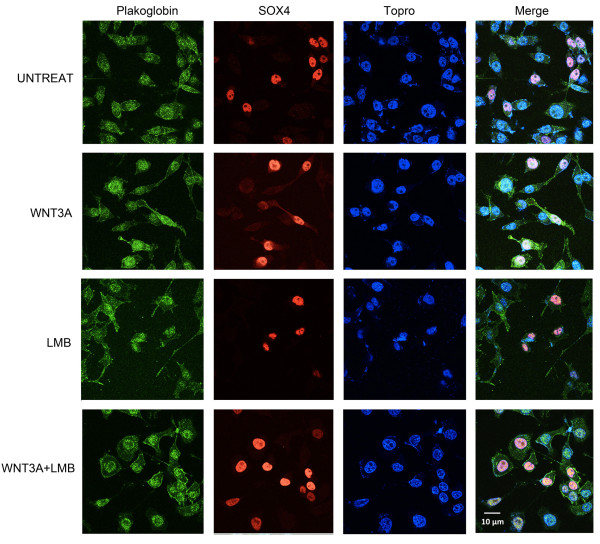
**Wnt signaling induces nuclear colocalization of SOX4 and plakoglobin**. Subcellular localization of plakoglobin and SOX4 were examined by confocal microscopy. PC3M HASOX4 stable cell line was treated with 100 ng/ml human recombinant WNT3A or 20 μM leptomycin b (LMB), or both WNT3A+LMB for 24 hr. The fields shown were visualized independently by confocal microscopy at the appropriate wavelength for plakoglobin (488) and SOX4 (543), and Topro (633) respectively, and then the three images were overlaid (Merge). Strong nuclear localization of plakoglobin was observed in the WNT3A+LMB treated cells that expressed HASOX4. Representative fields from these independent repeated experiments are shown. Plakoglobin localizes to the nucleus following WNT3A treatment, and this effect is strongly enhanced by LMB co-treatment, suggesting shuttling of plakoglobin into and out of the nucleus following WNT3A stimulation. Note that plakoglobin nuclear localization is much stronger in cells expressing HASOX4, suggesting SOX4 may facilitate plakoglobin nuclear import.

**Figure 3:**
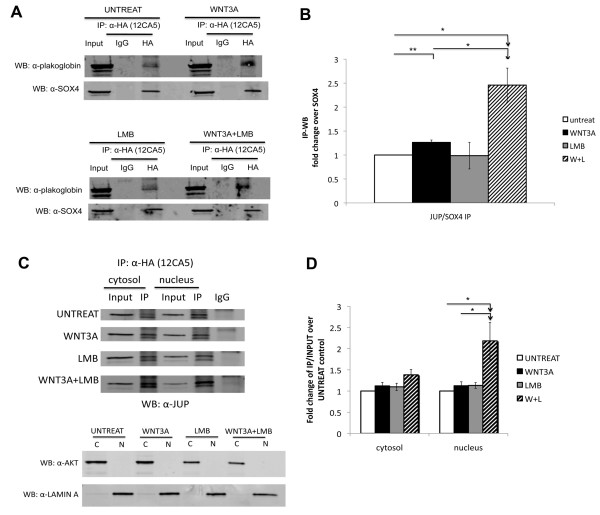
**Interaction between SOX4 and plakoglobin is affected by Wnt3A signaling**. *A*. LNCaP HASOX4 stable cell lines were treated with vehicle, Wnt3A, LMB, or Wnt3A + LMB and anti-HA immunoprecipitations were immunoblotted for the presence of plakoglobin. *B*, Quantitative analysis of co-IP and Western blots from panel A using the Odyssey^® ^Infrared Imaging System (Li-Cor Bioscience). Fold changes were calculated as the ratio of immunoprecipitated plakoglobin to SOX4 relative to the vehicle control panel (untreat). (n = 3, independent biological replicates performed on separate days; error bars represent SEM; *, p < 0.05; **, p < 0.01 for a one-sided paired t-test) *C*. (Upper) LNCaP HASOX4 stable cell lines were treated with vehicle, WNT3A, LMB, or WNT3A + LMB and anti-HA immunoprecipitations were performed using separate cytosolic and nuclear fractions. (Lower) Anti-AKT and anti-Lamin A antibodies were used as controls to examine markers of cytosolic and nuclear fractions, respectively, to confirm the purity of each fraction. *D*. Quantitative analysis of co-IP and Western blots from panel C.

When plakoglobin is present in desmosomes, it interacts with desmoglein and desmocollin, and when in adherens junctions it interacts with E-cadherin in the cytoplasmic component [15]. Recently, additional evidence has suggested that plakoglobin contributes a low level of transcriptional activity to the Wnt signal transduction cascade in the nucleus [19,20]. Although, it has been confirmed that SOX4 modulates Wnt signaling via interaction with β-catenin [5,23], the role of plakoglobin in Wnt signaling is still debated. To investigate where and under what conditions SOX4 and plakoglobin interact with each other, we used confocal microscopy to determine whether we could observe subcellular co-localization of HA-SOX4 and plakoglobin (Figure 2). After treatment of PC3M cells that stably-expressed HA-SOX4 with recombinant human WNT3A, we observed that the interaction between HA-SOX4 and plakoglobin was slightly increased in the nucleus. In contrast, this was not observed in the non-transfected adjacent cells. We observed the same phenomenon in the LNCaP-HA-SOX4 stable cell line (Additional file 1 Figure S1). Furthermore, when we treated with nuclear export inhibitor, leptomycin b (LMB) to inhibit nuclear export of SOX4 and plakoglobin, the co-localization was significantly increased compared to treating the cells with either WNT3A or LMB alone (Figure 2). To confirm these enhanced interactions, we performed co-immunoprecipitation under the same Wnt-induced conditions in LNCaP-HA-SOX4 cells (Figure 3A). The interaction in the whole cell lysate was quantified and significantly increased when we treated with WNT3A and LMB together (Figure 3B). In addition, to determine that SOX4 and plakoglobin interact in the nucleus, we prepared cytosolic and nuclear fractions to confirm the subcellular interaction (Figure 3C). Immunoblots against AKT and nuclear lamin were used as controls to demonstrate the purity of the nuclear and cytosolic fractions (Figure 3C). The quantitative results showed that the interaction in the nuclear but not cytosolic fraction was significantly increased in response to the WNT3A and LMB treatment (Figure 3D). These results show that SOX4 and plakoglobin physically interact in the nucleus of LNCaP cells.

### Wnt target genes and SOX4-target genes are affected by SOX4-plakoglobin interaction

**Figure 4:**
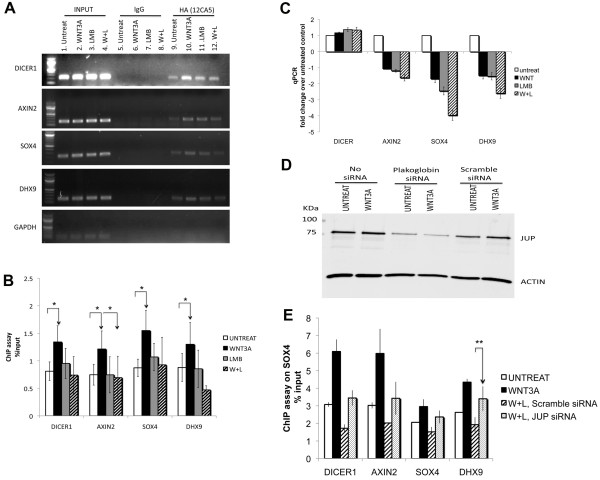
**SOX4 transcriptional activity is modulated by Wnt-induced interaction with plakoglobin**. *A*. ChIP assay of HA-SOX4 bound to the predicted SOX4-binding sites on *DICER1*, *SOX4*, *DHX9*, and *AXIN2 *promoters. *GAPDH *is shown as a negative control. *B*. Quantitative results of ChIP by realtime-PCR. SOX4 promoter occupancy is increased with Wnt treatment, but is strongly reduced by combined WNT3A+LMB treatment. (n = 4; error bars represent SEM; *, p < 0.05 for a one-sided paired t-test) *C*. SOX4 targets and Wnt downstream genes are inhibited after Wnt induction and LMB treatment. Realtime-PCR expression analysis of SOX4 direct targets and Wnt signaling downstream genes after 24 hrs of WNT3A and LMB treatment. *D*. siRNA directed against plakoglobin downregulates protein expression in LNCaP HA-SOX4 cell lines. Cells were harvested 48-hr post-transfection and Western blot were probed with anti-plakoglobin and anti-β-actin antibodies in the presence or absence of WNT3A treatment. *E*. Quantitative ChIP-qPCR assay of HA-SOX4 following plakoglobin knockdown. HA-SOX4 promoter occupancy is strongly reduced by combined WNT3A and LMB treatment but is partially restored when plakoglobin is knocked down by siRNA treatment. Control scrambled siRNA had no effect (n = 3; error bars represent SEM; *, p < 0.05 for a one-sided paired t-test).

To address the functional consequences of modulation of SOX4 transcriptional activity by the SOX4-plakoglobin complex, we tested expression of several genes including the Wnt target gene *AXIN2 *[29], as well as SOX4 targets *DICER1 *and *DHX9 *[23]. To characterize if SOX4 DNA binding activity is changed by Wnt-induced interaction with plakoglobin, we performed ChIP assays for *AXIN2*, *DICER1*, *DHX9*, and *SOX4 *(Figure 4A, B). Compared to untreated LNCaP HASOX4 cells, the binding of SOX4 to *AXIN2*, *DICER1*, and *DHX9 *promoters was increased after Wnt signaling was induced. However, SOX4 binding was decreased after treatment with both WNT3A and LMB. This difference indicates that increasing the interaction between SOX4 and plakoglobin could inhibit SOX4 binding activity to downstream targets and may inhibit SOX4 transcriptional activity. Quantitative realtime-PCR (qPCR) analysis of the LNCaP SOX4 stable cell line showed reductions in AXIN2, SOX4, and DHX9 upon co-treatment with WNT3A and LMB (Figure 4C). To determine whether the effects from combined WNT3A and LMB treatment were dependent on plakoglobin, we targeted plakoglobin by siRNA to determine whether reduced plakoglobin levels could rescue the effects we observed on SOX4 binding to target promoters. Transfection of LNCaP HA-SOX4 cells with plakoglobin siRNA or scrambled control siRNA resulted in approximately 50% knockdown of endogenous plakoglobin protein levels (Figure 4D). Furthermore, plakoglobin siRNA partially rescued SOX4 binding to target promoters by ChIP assay in the presence of WNT3A and LMB, while scrambled siRNA had no effect (Figure 4E). These data suggest that SOX4-plakoglobin interactions may interfere with SOX4-mediated transcription in response to Wnt signaling due to reduced promoter occupancy.

### SOX4-plakoglobin complex modulates β-catenin-mediated transcriptional activity

**Figure 5:**
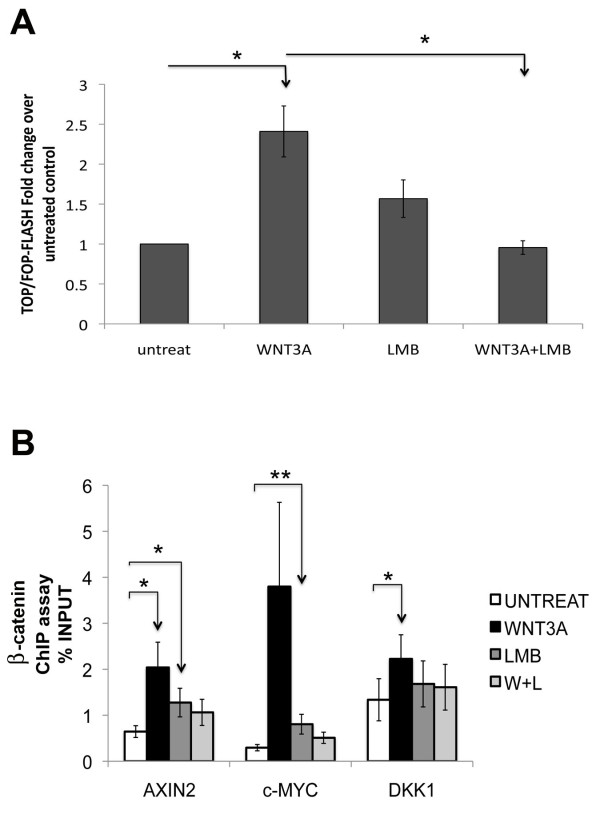
**Wnt signaling is downregulated by SOX4-plakoglobin complex**. *A*. Luciferase assay of LNCaP HASOX4 stable cell line co-transfected with 40 ng of TK-Renilla with either 500 ng of TOP-flash or FOP-flash plasmids. Luciferase activity was increased after Wnt inducement, but decreased in combined WNT3A+LMB treatment. (n = 3; error bars represent SEM; *, p < 0.05 for a one-sided paired t-test) *B*. Quantitative results of ChIP of β-catenin by realtime-PCR. β-catenin promoter occupancies of Wnt downstream targets, *AXIN2*, *c-MYC*, and *DKK1*, were increased with Wnt treatment, but is strongly reduced by combined WNT3A+LMB treatment. (n = 3; error bars represent SEM; *, p < 0.05; **, p < 0.01 for a one-sided paired t-test)

To evaluate whether SOX4-plakoglobin complex affects the transcriptional activity of β-catenin, we performed luciferase reporter assays with T cell factor (TCF) reporter plasmids containing wild type TCF binding sites (TOP-flash) or mutated TCF binding site (FOP-flash) [30] (Figure 5A). As expected, the TCF/β-catenin luciferase reporter was significantly increased after we induced Wnt signaling with recombinant WNT3A when compared to untreated cells. In contrast, co-treatment with WNT3A and LMB, strongly inhibited increases in luciferase activity back to baseline unstimulated levels, suggesting that WNT3A+ LMB-induced SOX4-plakoglobin complexes could compete with and inhibit the transcriptional activity of β-catenin. In addition, we performed anti-β-catenin ChIP assays on the *AXIN2, c-Myc*, and *DKK1 *promoters, and observed that occupancy of these promoters was stimulated by WNT3A, but that stimulation was inhibited by co-treatment with WNT3A and LMB (Figure 5B). These results suggest that β-catenin activity was affected by the SOX4-plakoglobin complex and that plakoglobin may compete with β-catenin binding to SOX4 and/or TCF/LEF in the nucleus.

## Discussion

While SOX4 expression is elevated in many malignancies and is tightly correlated with prostate cancer tumor grade, little is known of the mechanism by which SOX4 affects the progression of prostate cancer. Using LC-MS/MS proteomic analysis, we identified a novel SOX4 binding protein, junction plakoglobin, in LNCaP prostate cancer cells. We observed a physical association between SOX4 and plakoglobin with both epitope-tagged and endogenous SOX4. Although the interactions of SOX4 with plakoglobin were enhanced by co-treatment of WNT3A and LMB, we could detect this interaction using four different untransfected and unstimulated cell types (Figure 1F-I) treated with DNase I. In addition, confocal microscopy and co-immunoprecipitation demonstrated co-localization of SOX4 and plakoglobin in the nucleus when Wnt signaling was induced. ChIP assays showed the SOX4-plakoglobin complex affected SOX4 DNA binding activity to the *AXIN2*, *DICER1*, and *DHX9 *promoters that are Wnt signaling downstream genes and SOX4-associated targets. In addition, mRNA expression changes were detected in *AXIN2*, *DICER1*, and *DHX9 *by realtime-PCR. These data suggest that the SOX4-plakoglobin complex may inhibit Wnt signaling. Indeed, conditions that induced the increased interaction between SOX4 and plakoglobin caused β-catenin TOP-FLASH transcriptional activity to be downregulated and reduced occupancy of the *c-Myc *promoter by β-catenin.

**Figure 6:**
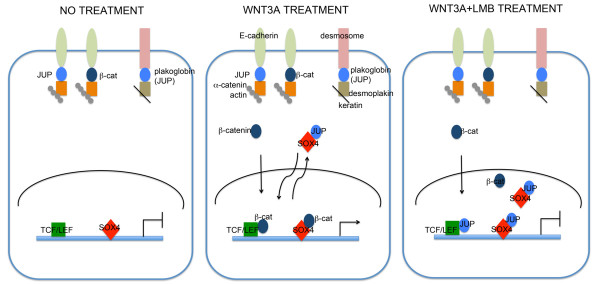
**Model depicting the role of SOX4 and plakoglobin in Wnt signaling regulation**. When cells are stimulated with WNT3A, Wnt-responsive genes are upregulated by nuclear localization of β-catenin and transcriptional activation by the β-catenin-TCF/LEF and β-catenin-SOX4 complexes. Plakoglobin then shuttles into the nucleus and is exported out of the nucleus with SOX4. When nuclear export is inhibited by LMB, plakoglobin competes with β-catenin for SOX4 binding, destabilizes SOX4 binding to target promoters, and inhibits transcription of target genes.

It is known that SOX4 can directly interact with β-catenin to enhance Wnt signaling [5,23], but mechanistic data remain very limited. We found that SOX4 interacts with plakoglobin in a WNT3A-dependent manner in our experimental cancer model. Our model not only supports the hypothesis that SOX4 may stabilize β-catenin [5], but also suggests a model in which SOX4 can modulate Wnt signaling by binding either β-catenin or plakoglobin (Figure 6). In this model, transcriptional responses to Wnt signaling are increased by SOX4-β-catenin interactions, and subsequently reduced by SOX4-plakoglobin interactions that facilitate nuclear export of SOX4. However, when nuclear export is inhibited, plakoglobin competes with β-catenin for binding to SOX4 and TCF/LEF factors, downregulating Wnt-responsive transcription and reducing SOX4-DNA binding. This model is supported by the observation that combination treatment with WNT3A and LMB enhanced SOX4-plakoglobin interactions, reduced TCF/β-catenin TOP-FLASH transcription, reduced SOX4-DNA binding and β-catenin DNA binding in ChIP assays, and reduced expression of SOX4 downstream targets (Figures 4 and 5). Consistent with our model, plakoglobin shows little transcriptional activity compared to β-catenin in cell lines that lack β-catenin [19].

Our confocal data show that plakoglobin nuclear localization is strongly enhanced by LMB co-treatment, suggesting shuttling of plakoglobin into and out of the nucleus following WNT3A stimulation. Although LMB is an artificial stimulus, others have shown that plakoglobin overexpression can lead to nuclear localization [31,32], and plakoglobin is overexpressed [33] and amplified [34] in several types of cancer. Moreover, plakoglobin nuclear localization was much stronger in cells expressing HASOX4, suggesting cytoplasmic SOX4 may facilitate plakoglobin nuclear import. Thus, SOX4 may induce nuclear import of plakoglobin in response to WNT3A while plakoglobin destabilizes SOX4 from DNA binding, facilitating nuclear export of SOX4. It is as yet unclear what signals or modifications might tip the balance between nuclear import and export of plakoglobin-SOX4 complexes, or whether shuttling might be constitutive.

The role of plakoglobin during cancer progression is still controversial. When plakoglobin is overexpressed, it induces cell migration and mobility in HCT116 cells, suggesting that plakoglobin may have some oncogenic effects [31]. In contrast, several reports have demonstrated that plakoglobin has a tumor-suppressive effect that inhibits tumor cell growth [27,35]. The tumor suppressive activity of plakoglobin may be via nuclear translocation to antogonize β-catenin binding to TCF/LEF proteins in keratinocytes [36], supporting our model that plakoglobin competes with SOX4-β-catenin interactions in the nucleus.

## Conclusion

In summary, we have demonstrated that SOX4 interacts with plakoglobin in a Wnt-dependent manner in LNCaP cells and that this complex may function to inhibit Wnt signaling. Additional studies will be required to elucidate the detailed mechanisms by which SOX4-plakoglobin interactions may affect Wnt signaling, but the role of the SOX4-plakoglobin complex provides novel insights into the role of SOX4 in Wnt signaling and prostate cancer progression.

## Authors' contributions

YHL performed the immunopurifications of SOX4, the ChIP assays, co-IPs, luciferase assays, and helped write the manuscript. JC cloned SOX4 into the pREP4-BLRPwt-IRES-BirA-XL9 plasmid and performed co-IPs. DC performed the proteomics analysis. MEF and KDB performed immunofluorescence confocal microscopy. JP advised on protein purification, supervised the proteomics analysis, and edited the manuscript. AN supervised the confocal microscopy and edited the manuscript. CSM conceived the study, participated in its design and coordination, and co-wrote the manuscript. All authors read and approved the final manuscript.

## Supplementary Material

Additional file 1 **Figure.S1 Wnt signaling induces nuclear colocalization of SOX4 and plakoglobin**. Subcellular localization of plakoglobin and SOX4 were examined by confocal microscopy. LNCaP HASOX4 stable cell line was treated with 100 ng/ml human recombinant WNT3A or 20 μM leptomycin b (LMB), or both WNT3A+LMB for 24 hr. The fields shown were visualized independently by confocal microscopy at the appropriate wavelength for plakoglobin (488) and SOX4 (543), and Topro (633) respectively, and then the three images were overlaid (Merge). Strong nuclear localization of plakoglobin was observed in the WNT3A+LMB treated cells that expressed HASOX4. Representative fields from these independent repeated experiments are shown. Plakoglobin localizes to the nucleus following WNT3A treatment, and this effect is strongly enhanced by LMB co-treatment, suggesting shuttling of plakoglobin into and out of the nucleus following WNT3A stimulation. Note that plakoglobin nuclear localization is much stronger in cells expressing HASOX4, suggesting SOX4 may facilitate plakoglobin nuclear import. Click here for file
