# Supplementary figures and images for: SOX4 interacts with plakoglobin in a Wnt3a-dependent manner in prostate cancer cells
Source: BMC Cell Biol. 2011 Nov 19;12:50. doi: 10.1186/1471-2121-12-50 (PMC3227594; doi:10.1186/1471-2121-12-50)

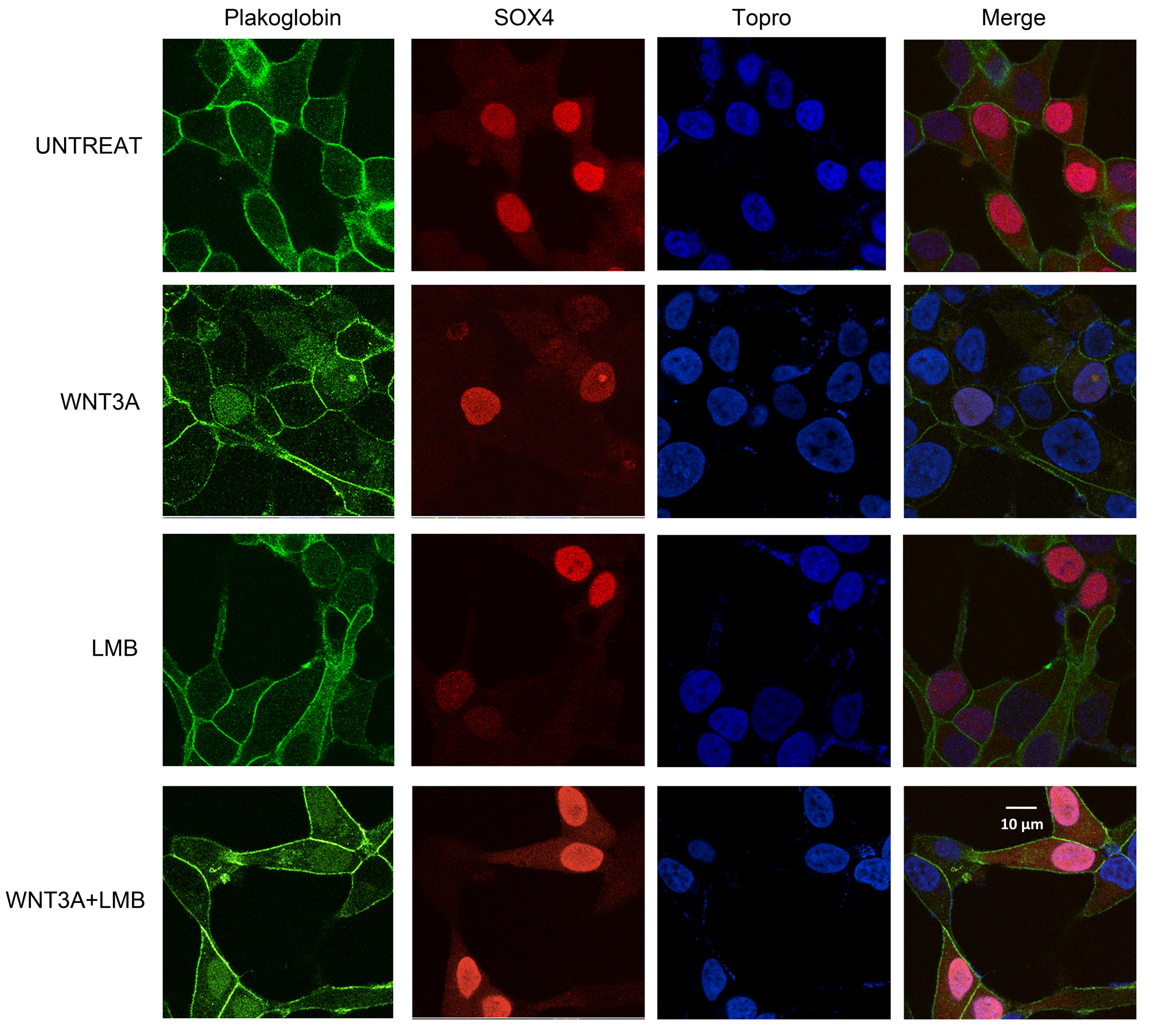

Supplement: Additional file 1 — Figure.S1 Wnt signaling induces nuclear colocalization of SOX4 and plakoglobin. Subcellular localization of plakoglobin and SOX4 were examined by confocal microscopy. LNCaP HASOX4 stable cell line was treated with 100 ng/ml human recombinant WNT3A or 20 μM leptomycin b (LMB), or both WNT3A+LMB for 24 hr. The fields shown were visualized independently by confocal microscopy at the appropriate wavelength for plakoglobin (488) and SOX4 (543), and Topro (633) respectively, and then the three images were overlaid (Merge). Strong nuclear localization of plakoglobin was observed in the WNT3A+LMB treated cells that expressed HASOX4. Representative fields from these independent repeated experiments are shown. Plakoglobin localizes to the nucleus following WNT3A treatment, and this effect is strongly enhanced by LMB co-treatment, suggesting shuttling of plakoglobin into and out of the nucleus following WNT3A stimulation. Note that plakoglobin nuclear localization is much stronger in cells expressing HASOX4, suggesting SOX4 may facilitate plakoglobin nuclear import. [file 1471-2121-12-50-S1.JPEG]
